# Seasonal Variation in Disease

**Published:** 1898-06-25

**Authors:** 


### SEASONAL VARIATION IN DISEASE.

The *Lancet* very appropriately speculates as to whether at such a time as the present, when, "with the advent of summer, vegetation is bursting forth and clothing the earth with its rich green mantle," those minute forms of vegetable life on which zymotic diseases depend are also undergoing growth and development in their natural habitat, wherever that may be. Taking up this line of thought the writer of the article in question, after an examination of the seasonal curves of disease mortality, comes to the conclusion that "there is but little evidence to support a view that the summer growth of vegetation as it manifests itself to us is accompanied by any great multiplication of pathogenic organisms." Nor may we add was it likely that such should be the case. The part played by the sort of vegetation that clothes the earth with its green mantle is so different from that played by pathogenic microbes —the one constructional, deriving, if one may so say, the very ingredients for the production of living matter from the energy of the sun's rays, the other destructional and dependent on the energy already provided by pre-existing life—that it is not to be expected that sunshine, so essential to the one, should be of equal or even of parallel importance to the other. Nevertheless, certain factors which go to produce the sort of weather which promotes verdure have also much influence upon the growth of many pathogenic organisms. As an example we may mention heat, which, both directly and in a secondary way, by the production of decomposition, favours the saprophytic growth of many of the germs of disease. Again, by altering the diet of man [the fruitful season induces in him a certain predisposition to some infections, so that although there is no parallelism between the growth of trees and verdure and that of pathogenic organisms, there is occasionally a coincidence between them. What is spoken of as the seasonal variation of disease is, indeed, the product of many factors besides those which may be expressed under the head of weather; and while some of these have to do with "natural causes," others are artificial and are related purely to the habits of the community under discussion. But the subject of seasonal variation is a very complex one, and as showing how far from simple it is we may allude to the very abstruse question of seasonal variation not in the prevalence, but in the fatality of certain diseases. The very striking figures collected by Mr. Shirley Murphy, and published in his Annual Reports to the London County Council, show that, whatever may be the conditions which favour a widespread epidemic, say, of scarlet fever, they are not the same conditions which favour a high fatality (case mortality). It would, in fact, appear that the virulence of such cases as do occur is greatest just at the time when the number of cases is least. Many explanations may be suggested, but there the fact is; and it alone is sufficient to show how wide is the subject of seasonal variation, and how little it can be explained by the more obvious peculiarities of "the seasons" as they are usually under stood.

